# Type I IFN receptor blockade by anifrolumab reduces cutaneous lupus in monogenic SLE

**DOI:** 10.1111/ddg.15806

**Published:** 2025-06-26

**Authors:** Julian Steininger, Christine Wolf, Min Ae Lee‐Kirsch, Claudia Günther

**Affiliations:** ^1^ Department of Dermatology Medizinische Fakultät Carl Gustav Carus Technische Universität Dresden Dresden Germany; ^2^ Department of Pediatrics Medizinische Fakultät Carl Gustav Carus Technische Universität Dresden Dresden Germany; ^3^ University Centre for Rare Diseases University Hospital Carl Gustav Carus Technische Universität Dresden Dresden Germany; ^4^ German Center for Child and Adolescent Health (DZKJ) partner site Leipzig/Dresden Dresden Germany; ^5^ Department of Dermatology Eberhard Karls University Tübingen Germany

Dear Editors,

We report the complete remission (CR) of cutaneous lupus erythematosus (CLE) in a 24‐year‐old man with early‐onset lupus caused by a biallelic C1QC mutation,^1^ achieved under treatment with the interferon‐alpha/beta receptor (IFNAR) antagonist anifrolumab.

Monogenic deficiencies in C1 components (C1QDef) are extremely rare with only 74 reported cases.^2^ They are strongly associated with increased expression of type I interferon (IFN) and type I IFN‐stimulated genes (ISGs), which drive an innate immune response against self, resulting in a lupus‐like phenotype. The symptoms in patients with C1QDef typically manifest in early childhood and, unlike in sporadic systemic lupus erythematosus (SLE), usually exhibit a poor response to standard SLE treatments. Therapies involving regular fresh frozen plasma or hematopoietic stem cell transplantation carry a high risk of complications. In addition to significant morbidity, C1QDef is also associated with increased mortality.^3^

Our patient suffered from early onset CLE‐like manifestations, with the first cutaneous symptoms noticed at the age of 4 (10/2003), for which he was treated with hydroxychloroquine (Figure 1). In April 2004, proteinuria, increased rheumatoid factors, and anti‐nuclear, anti‐cardiolipin, and anti‐SSA antibodies appeared, resembling manifestations of SLE. Two years later, the patient began to experience fluctuating pain and swelling in the elbow joints. Additionally, borderline levels of anti‐double‐stranded DNA antibodies were detected, prompting the initiation of therapy with azathioprine and prednisolone. Due to severe renal deterioration with macrohematuria and proteinuria (12/2008), the patient was switched to mycophenolate (MMF) in combination with hydroxychloroquine.

**FIGURE 1:**
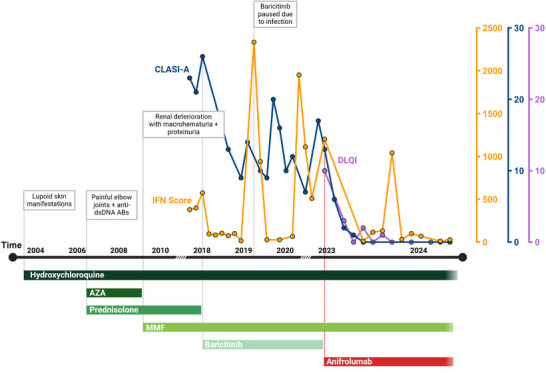
Timeline of a patient with biallelic C1QC mutation. Time course of clinical presentation and medications used shown below. The orange line indicates the patient's interferon (IFN) scores. The blue line indicates the patient's CLASI activity (CLASI‐A) score. The purple line indicates the patient's score in the Dermatology Life Quality Index (DLQI). IFN scores were determined by measuring the mRNA expression of seven ISGs (IFI27, IFI44, IFI44L, IFIT1, ISG15, SIGLEC1, and RSAD2) normalized to GAPDH and HPRT1 and compared to a healthy cohort in peripheral blood mononuclear cells.^1^ *Abbr*.: AZA, azathioprine; MMF, mycophenolate mofetile

From 2015 onwards, the SLE flares, including acute and discoid skin lesions (Figure 2), required monthly pulse therapy with prednisolone. Mutation analyses, performed after the first presentation in our clinic (02/2018), revealed a homozygous mutation in the *C1QC* gene (c.205C>T, p.Arg69Ter).^1^ Because of inadequate disease control, the JAK inhibitor (JAKi) baricitinib was added to the treatment. The drug was well tolerated and symptoms improved.^1^ However, fluctuating discoid plaques remained, especially on the face, and *CLE Disease Area and Severity Index Activity* (CLASI‐A) scores^4^ did not normalize completely (Figures 1, 2). Interestingly, the IFN score in blood declined significantly but this did not correlate with CR of CLE. Therefore, treatment was switched from baricitinib to infusions of 300 mg anifrolumab every 4 weeks. Anifrolumab is an IgG1κ monoclonal antibody that inhibits the binding of all type I IFN subtypes to their single common receptor. Several case series and clinical trials have demonstrated its efficacy in the treatment of CLE lesions.^5^, ^6^ The drug was most effective in patients with elevated type I IFNs,^7^, ^8^ and has been shown to substantially reduce the type I IFN signature in SLE patients.^9^

**FIGURE 2:**
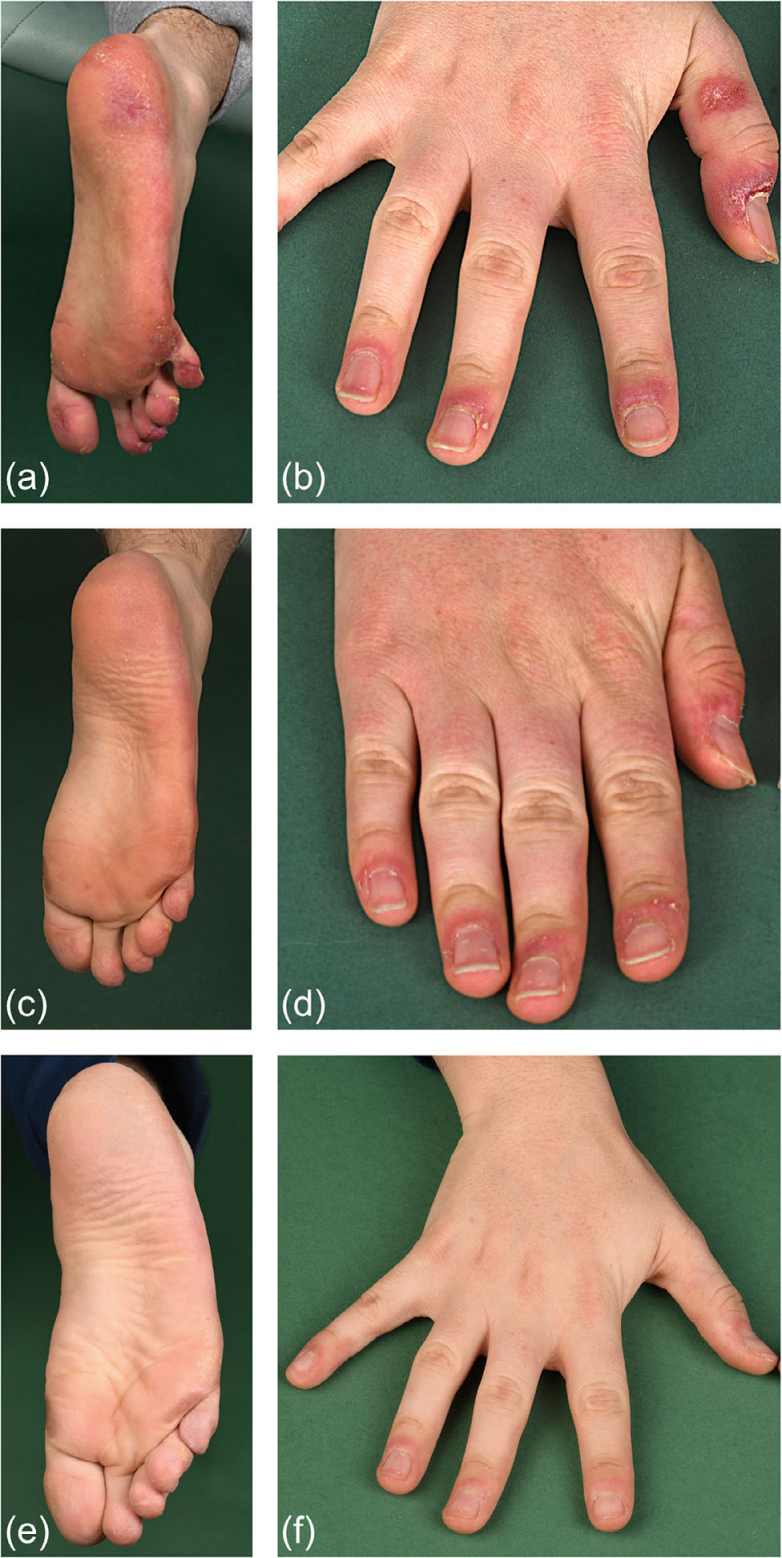
Improvement of disease in a patient with biallelic *C1QC* mutation during treatment with the interferon‐alpha/beta receptor (IFNAR) antagonist anifrolumab. (a, b) Poorly defined livid‐erythematous plaques on the feet causing pain when walking; acral erythematous erosions and ulcerations on the hands. (c, d) Three months after initiation of anifrolumab, with improvement of skin manifestations on the feet and hands. (e, f) Almost complete resolution of skin lesions on the feet and hands after one year of therapy with anifrolumab.

A rapid clinical response was observed following the first infusion with anifrolumab: previously refractory skin lesions improved by 50% within one month, as measured by CLASI‐A scores, and resolved almost completely within 12 months of treatment (Figures 1, 2). Importantly, the highly therapy‐resistant chilblain like lesions and vasculitis of the fingers and palms responded to the IFN blockade (Figure 2). This underscores the rarely reported observation of potential efficacy of anifrolumab in chilblain lupus.^10^ The patient's quality of life also improved (Figure 1).

The patient did not report any severe adverse events. However, he experienced a significant increase in the frequency of mild respiratory infections as well as a first episode of thoracic herpes zoster, which prompted us to extend the treatment intervals to 6 weeks. This was tolerated but accompanied by recurrent flares of CLE already after 2 months. Consequently, the previous treatment interval of 4 weeks was readopted.

Adding anifrolumab to baseline therapy with hydroxychloroquine and MMF resulted in superior control of CLE compared to baricitinib (median CLASI‐A baricitinib 13.00, median CLASI‐A anifrolumab 1.00; p  =  0.0009).^6^, ^7^, ^11^, ^12^ In line with our findings, Triaille et al. also reported insufficient disease control with JAKi treatment.^3^ This may be explained by the fact that C1QDef‐patients exhibit similarly high IFN signatures as in classic interferonopathies, which could account for the superior outcomes achieved with direct IFNAR‐blockade. In contrast, JAKi impact multiple signaling pathways as JAKs are involved in the signal transduction of various cytokines. Consequently, JAKi may provide a broader, albeit potentially less complete, blockade of the affected type I IFN pathway. However, further studies are required to fully elucidate the long‐term effects of both drugs.

Given the absence of head‐to‐head studies between JAKi and anifrolumab, it remains possible that anifrolumab may also demonstrate superior pharmacological efficacy on cutaneous inflammation. The improved clinical response, however, is not reflected in the type‐I IFN score in the blood, which decreased to a comparable extent under both baricitinib and anifrolumab. Therefore, documentation of the CLE maifestations using verified clinical scores is important in clinical trials in SLE and their correlation with ISG levels in blood needs to be further validated.

## FUNDING

This work was supported by the *Deutsche Forschungsgemeinschaft* (DFG, German Research Foundation), grant TRR237 369799452/404458960 to C.G. M.L.‐K. is supported by DFG grants CRC237 369799452/B21, CRC237 369799452/A11, CRC369 501752319/C06 and by grants of the *German Federal Ministry of Education and Research* (BMBF) 01GM2206C (GAIN) and 01GL2405H (DZKJ). C.W. is supported by the DFG grant CRC237 369799452/A06.

## CONFLICT OF INTEREST STATEMENT

C.G. has received honoraria for scientific lectures, participation on advisory boards, and research funding from AstraZeneca, GSK, Boehringer, Almirall, and Janssen. The other authors declare that the research was conducted in the absence of any commercial or financial relationships that could be construed as a potential conflict of interest.
